# Preventible Social Ills

**Published:** 1912-01-27

**Authors:** 


### PREVENTIBLE SOCIAL ILLS.

### The Spirit of Pauperism.

The true pauper spirit is grounded on irresponsibility. It is natural to man even from babyhood to struggle for the means of subsistence and look after himself.

### Influence of the Family.

In healthy family life he learns to look after others also, and to subordinate his own cravings to the call of those more feeble than himself. His character becomes strengthened by calls upon him for service and thus to a sense of responsibility towards himself is added the sense of reponsibility towards employer, parents, wife, or family. This subtle tendency in a man's nature to respond to the calls made upon him through life is the basis on which is built the prosperity of the nation. It is futile to attempt to instil it by verbal teaching alone, though it soaks into the most unpromising natures when they are placed under favourable conditions. It comes impalpably, and, alas, it flees at a touch. No sooner is a man forced to resort to the help of his neighbours, extorted from them through the agency of the rates, than he may become irresponsible.

### Variations in London Pauperism.

If pauperism were a natural result of poverty it would follow that in the poorest parts of London, where to keep a servant is evidence of exceptional wealth, the ratio of paupers per 1,000 of the population would be highest. Yet taking the figures for last December in Whitechapel the ratio was but 22.1, and in Bethnal Green but 23.5, as contrasted with the wealthy locality of St. George's, where the ratio was 26.3, and of St. Marylebone with a ratio of 28.4.

To accept the easy inference that in those parts of London where pauperism is reduced to a minimum the Guardians are deaf to the call of distress would be to ignore facts and overlook public work of the most valuable, though least conspicuous, character. The pauper as we know him is the product of a slovenly ill-organised system of assistance. When he first applies to the Guardians he may be destitute but he is not a pauper. To reinstate him in a position wherein he can look after himself and those who depend upon him is the vital matter for the community to undertake in their own interests as well as in his.

This difficult task is being undertaken with splendid success in localities where it has been possible to secure the co-operation of a trained band of voluntary workers. In Whitechapel, for instance, the outdoor list of paupers was reduced in ten years from 5,300 to 128 and stands now at the lowest figure for the whole of London. This result has been attained by intelligent study of the circumstances leading to destitution in each individual case, followed up by joint action on the part of many admirable voluntary societies. The Relieving Office, instead of being a bureau for the manufacture of paupers has been turned into a social force of the strongest possible character for its abolition.

### Voluntary Agencies Indispensable.

The urgent need of the moment is for increased co-operation in all localities between the officials of the Poor Law and the voluntary agencies. Whatever form the new organisation of the Poor Law may take, this is its most vital necessity. In order to cut at the root of that helpless submission to want which is the antithesis of a healthy struggle for existence, the men who have gone under owing to the pressure of social conditions outside their own volition, need to be helped to feel and use their own strength.

In scientific language the right "stimulus" must be applied. Under the Poor Law free will and initiative are stifled and the futile longing for escape, a longing which the pauper frequently gratifies, always to drift back to the workhouse, takes the place of healthy endeavour. To treat each potential pauper as an individual with latent possibilities of doing good work in the world is only possible under an organisation reinforced by people inspired with the enthusiasm for humanity. If throughout London the myriad strands of altruistic endeavour were linked together into a coherent body of trained workers, aiming at the extinction of pauperism, the manufactured article, turned out from the workhouses to drift in and out at will, could be made to disappear within ten years, as it has disappeared in Whitechapel.

It was the great blunder of the Poor Law that it made a sharp severance between the philanthropical who help the needy from love, and the ratepayer who helps them because he is compelled to do so by law.

It is not too late to repair the mischief, and the parishes in which voluntary workers have taken upon them the duties of Guardians and leavened the administration from within should serve as object-lessons in any revision of regulations. Their difficulties, their triumphs, and the obstacles which have proved too immovable even for their zeal, should point the way to a new and fruitful co-operation between business and sentiment in the highest senses of both.

### Disastrous Influence of Improved Poor Law Methods.

There is a general feeling among the public, due to the expensive buildings and the real zeal of Poor Law authorities, that there is little fault to be found with those aspects of the work which deal with the infirm, the sick, and the children. Certainly there has been amelioration, and abuses are more watchfully dealt with than formerly. But even were it possible to attain a far higher standard in dealing with the young and the sick, the pauperising effects of entrusting their care to the same authority which must deal with the vagrant would be aggravated rather than lessened.

